# Copper/Zinc-Modified Palygorskite Protects Against *Salmonella* Typhimurium Infection and Modulates the Intestinal Microbiota in Chickens

**DOI:** 10.3389/fmicb.2021.739348

**Published:** 2021-12-09

**Authors:** Chaozheng Zhang, Dawei Yao, Zenan Su, Huan Chen, Pan Hao, Yun Liao, Yiwen Guo, Deji Yang

**Affiliations:** College of Veterinary Medicine, Nanjing Agricultural University, Nanjing, China

**Keywords:** copper/zinc-modified palygorskite, *Salmonella typhimurium*, mucosal barrier, chickens, intestinal microbiota

## Abstract

Palygorskite (Pal), a clay nanoparticle, has been demonstrated to be a vehicle for drug delivery. Copper has antibacterial properties, and zinc is an essential micronutrient for intestinal health in animals and humans. However, whether copper/zinc-modified Pal (Cu/Zn-Pal) can protect chickens from *Salmonella enterica* subsp. *enterica* serovar Typhimurium (*S.* Typhimurium) infection remains unclear. In this study, three complexes (Cu/Zn-Pal-1, Cu/Zn-Pal-2, and Cu/Zn-Pal-3) were prepared, and Cu/Zn-Pal-1 was shown to be the most effective at inhibiting the growth of *S.* Typhimurium *in vitro*, whereas natural Pal alone had no inhibitory effect. *In vivo*, Cu/Zn-Pal-1 reduced *S.* Typhimurium colonization in the intestine of infected chickens and relieved *S.* Typhimurium-induced organ and intestinal mucosal barrier damage. Moreover, this reduction in *Salmonella* load attenuated intestinal inflammation and the oxidative stress response in challenged chickens. Additionally, Cu/Zn-Pal-1 modulated the intestinal microbiota in infected chickens, which was characterized by the reduced abundance of Firmicutes and the increased abundance of Proteobacteria and Bacteroidetes. Our results indicated that the Cu/Zn-Pal-1 complex may be an effective feed supplement for reducing *S.* Typhimurium colonization of the gut.

## Introduction

*Salmonella*, a common zoonotic enteric pathogen, poses a serious threat to global public health (Xu et al., 2018; Zhao X. et al., 2020). *Salmonella* is transmitted to humans through the food chain *via* contaminated poultry meat, eggs, and water, which is the primary cause of salmonellosis outbreaks (Guerrero et al., 2020; Xu Y. et al., 2020). Furthermore, *Salmonella* infection in chickens causes intestinal microbiota dysfunction, inflammatory diarrhea, compromised production performance, and even death, leading to huge economic losses to the poultry industry (Wang et al., 2019). In the past, antimicrobial therapy was widely administered through feed to aid in the elimination of severe *Salmonella* infection (Hur et al., 2012). At present, however, antibiotics are prohibited in feed due to the increase in resistance among *Salmonella* strains and antibiotic residues in poultry products (Hur et al., 2012; Peng et al., 2018; Wang et al., 2019). Therefore, it is necessary to explore new effective medications to protect against *Salmonella* infection in broilers.

Clay nanoparticles play a variety of roles in medicine as antacids, gastrointestinal protectors, antidiarrheic agents, and other active substances (Mousa et al., 2018). Palygorskite (Pal), formerly known as attapulgite, is one type of clay nanoparticle with the theoretical formula Si_8_O_20_(Mg,Al,Fe)_5_(OH)_2_(OH_2_)_4_•H_2_O (Xu N. et al., 2020). Pal is characterized by a large surface area, great cation exchange capacity, strong absorptive capacity, and slow releasing properties, and is commonly used as a vehicle for antimicrobial substances (Li et al., 2014; Lei et al., 2017). It has been reported that natural clay minerals have no antibacterial activity themselves, but the modified clay minerals adsorb and then kill bacteria when the clay minerals bind to antibacterial substances (Rapacz-Kmita et al., 2017; Mousa et al., 2018). Previous research has shown that a ginger essential oil/Pal (GEO/Pal) composite had higher anti-*Escherichia coli* and anti-*Staphylococcus aureus* capability than GEO alone due to the bacteria-absorbent activity of Pal and the bactericidal action of GEO (Lei et al., 2017; Xu N. et al., 2020). Furthermore, a recent study indicated that compared with silver nanoparticles stabilized with cashew gum (AgNPs-CG), a Pal/AgNPs-CG nanocomposite enhanced the effects against *E. coli* and *S. aureus*. The large surface area of Pal increased the contact between the Pal/AgNPs-CG nanocomposite and bacteria, enhancing the antibacterial action of AgNPs-CG (Araújo et al., 2020). Additionally, a study reported that dodecyl triphenyl phosphonium bromide/Pal (DTP/Pal) hybrids, combining the advantages of Pal and DTP, displayed long-term antibacterial activity (Cai et al., 2013). Modified Pal exhibits great cation exchange capacity, a large specific surface area, and colloid properties that give rise to optimum adsorption of bacteria, and then uses the antibacterial properties of antibacterial materials for sterilization, which is similar to the antibacterial mechanism of modified montmorillonite (Khurana et al., 2015). Although the above Pal complexes have been shown to have bactericidal action *in vitro*, whether they protect against pathogen infection *in vivo* and their protective mechanism remain to be elucidated.

Copper has good antibacterial capacity and is widely used in daily life to reduce the spread of pathogens (Peters et al., 2018). Copper also is an essential trace element in animals and humans and plays a vital role in critical enzyme systems to maintain good health (Ghosh et al., 2019). Zinc is necessary for maintaining the structure and barrier function of the intestine (Shao et al., 2017). Moreover, pharmacological doses of zinc can alleviate intestinal permeability and diarrhea in weaning piglets (Shao et al., 2017).

A preliminary study in our laboratory found that copper-modified Pal was effective in preventing and treating diarrhea caused by *Salmonella enterica* subsp. *enterica* serovar Typhimurium (*S.* Typhimurium) in mice (Yao et al., 2017). Therefore, in this study, we aimed to investigate the protective effects of copper/zinc-modified Pal (Cu/Zn-Pal) pretreatment on chickens infected with *S.* Typhimurium and explore the underlying protective mechanism.

## Materials and Methods

### Preparation of Cu/Zn-Pal Complexes

The Cu/Zn-Pal complexes were prepared *via* an ion-exchange reaction. Briefly, 10 g of Pal (Jiangsu Shenlite Biotechnology Co., Ltd., Jiangsu, China) was dispersed in 200 ml of 0.15 mol/L CuSO_4_ solution, 0.15 mol/L ZnCl_2_ solution, 0.15 mol/L CuSO_4_, and ZnCl_2_ mixed solution (to mol ratios of Cu^2+^ to Zn^2+^ of 4:1, 1:1, 1:4) to prepare Cu-Pal, Zn-Pal, Cu/Zn-Pal-1, Cu/Zn-Pal-2, and Cu/Zn-Pal-3, respectively. The above five solutions were stirred vigorously for 6 h at 90°C and 200 r/min. Then, the precipitate was washed repeatedly with distilled water, until there was no blue flocculent precipitation or white precipitation on addition of NaOH or AgNO_3_ solution, respectively, indicating that there was no CuSO_4_ or ZnCl_2_ remaining in the wash solution (Ma et al., 2015; Martina et al., 2019). Finally, Cu-Pal, Zn-Pal, Cu/Zn-Pal-1, Cu/Zn-Pal-2, and Cu/Zn-Pal-3 were obtained after drying at 105°C, crushing and filtering through a 200-mesh strainer. The copper and zinc contents of the complexes were detected by inductively coupled plasma-optical emission spectrometry (Optimal 2100DV, Perkin Elmer Instruments, Waltham, MA, United States).

### Anti-*Salmonella* Activity of Cu/Zn-Pal *in vitro*

The anti-*Salmonella* activity of the complexes was evaluated using the agar diffusion method. The *S.* Typhimurium NJS1 strain (pig isolate) was cultivated in Mueller–Hinton broth (Oxoid, Basingstoke, United Kingdom) overnight at 37°C. The following day, the bacterial suspension was adjusted to 0.5 McFarland standard with phosphate-buffered saline (PBS; Solarbio, Beijing, China) and was then applied to Mueller–Hinton agar (Oxoid) plates using sterile cotton swabs. Then, the plates were perforated with 6-mm diameter holes, in which 50 μl of 0.1 g/ml of the complexes (Pal, Cu-Pal, Zn-Pal, Cu/Zn-Pal-1, Cu/Zn-Pal-2, and Cu/Zn-Pal-3) was placed, and the plates were incubated for 24 h at 37°C. The diameter of the inhibition zone was measured for each complex. Three independent experiments were carried out, and each experiment was repeated three times.

The minimum bactericidal concentration (MBC) of the complexes was assessed by the colony counting method. The different concentrations of complexes (Pal, Cu-Pal, Zn-Pal, Cu/Zn-Pal-1, Cu/Zn-Pal-2, and Cu/Zn-Pal-3) ranging from 2 to 64 mg/ml were dispersed in 10 ml of Luria–Bertani (LB) broth (10 g/L tryptone, 5 g/L yeast extract, 5 g/L NaCl; Oxoid), in which 100 μl of 1 × 10^7^ colony forming units (CFU)/ml *S.* Typhimurium was inoculated. The mixtures were shaken for 24 h at 37°C and 180 rpm/min. Then, 100 μl of the cultures was swabbed onto LB agar plates for colony counting. The concentration of each complex that completely inhibited visible colony formation on plates was considered the MBC. Three experiments were performed independently, and each experiment was carried out in triplicate.

The growth curve of *S.* Typhimurium was also measured using the colony counting method. Briefly, 100 μl of 1 × 10^7^ CFU/ml *S.* Typhimurium and different concentrations of Cu/Zn-Pal-1 (0 × MBC, 0.5 × MBC, 1 × MBC, and 2 × MBC) or different substances (Cu/Zn-Pal-1, Cu^2+^/Zn^2+^, Cu^2+^/Zn^2+^ + Pal, and Pal) were added to 10 ml of fresh LB medium, which was shaken at 37°C and 180 rpm/min. Dilutions of the cultures were applied to LB agar plates at 2-h intervals, and the plates were cultured overnight at 37°C for colony counting. The results are shown as the mean and standard deviation (error bars) from triplicate experiments.

### Animal Experiment Design and Sample Collection

Experiments involving chickens were approved by the Institutional Animal Care and Use Committee of Nanjing Agricultural University (Nanjing, China). Newborn Sanhuang chicks (*n* = 75) were randomly assigned to five groups (*n* = 15/group): (1) control (CON), (2) Cu/Zn-Pal-1 (CZP), (3) *Salmonella* (SAL), (4) Cu/Zn-Pal-1 + *Salmonella* (CZPS), and (5) chloramphenicol + *Salmonella* (CHL). Chicks in the CON and SAL groups were fed with broiler mixed feed 601 (consisting of maize, wheat, soybean meal, limestone, crude protein, calcium, and lysine) (Anhui New Hope Feed Co., Ltd., Hefei, China) for 20 days, chicks in the CZP and CZPS groups were fed with broiler mixed feed 601 supplemented with 5 g/kg Cu/Zn-Pal-1 for 20 days, and chicks in the CHL group were fed with broiler mixed feed 601 supplemented with 0.1% w/w chloramphenicol for 20 days. With the exception of the CON and CZP groups, all chicks were orally administered *S.* Typhimurium (2 × 10^9^ CFU) suspended in 0.5 ml of PBS on day 4.

The body weight of the chicks was measured every 2 days. In addition, the feces of the broiler chickens were collected on day 20, diluted with PBS, and then plated onto *Salmonella–Shigella* agar (Qingdao Hope Bio-Technology Co., Ltd., Qingdao, China) for enumerating *S.* Typhimurium colonization in the chick feces. In addition, blood was obtained from the jugular vein of each chick, and serum was separated by centrifugation at 1,000 × *g* for 10 min at 4°C and then stored at −20°C. Then, the chickens were euthanized *via* cervical dislocation, and tissues including the liver, spleen, intestine, and ceca were collected aseptically for *S.* Typhimurium enumeration. Intestinal digesta samples were obtained quickly and stored at −80°C for intestinal microbial composition analysis. A fraction of the duodenum of each chicken was fixed in Carnoy’s fluid (Wuhan Servicebio Biotechnology Co., Ltd., Wuhan, China) for histological analysis, and the ileum was harvested and stored at −80°C for real-time PCR, ELISA, and oxidative stress analysis.

### Determination of *S.* Typhimurium Colonization in the Feces and Tissues

The feces and tissues, including the liver, spleen, intestine, and ceca, were homogenized and then diluted 1:10 with sterile PBS. For each dilution, 100 μl of suspension was coated onto *Salmonella–Shigella* agar plates, which were incubated for at least 24 h at 37°C. The CFU per plate were enumerated and then *S.* Typhimurium colonization in the feces and tissues was calculated. Data were collected from three independent samples from each group, and each sample was performed in triplicate.

### Histological Analysis

For histological analysis, a small segment of the duodenum was fixed in Carnoy’s fluid, dehydrated and embedded in paraffin (*n* = 6/group). Paraffin sections of 5 μm were stained with hematoxylin–eosin and periodic acid–Schiff. Images of the intestinal villi and crypt were captured using a Leica microscope (Leica Microsystems, Mannheim, Germany). Intestinal villus height and crypt depth were measured and analyzed for at least 50 villi or crypts/chick for each paraffin section using the Image J software (NIH ImageJ System, Bethesda, MD, United States) (Hou et al., 2018).

### Intestine and Serum Index Determination

The collected ileum samples were homogenized and centrifuged. The levels of secretory immunoglobulin A (sIgA), immunoglobulin G (IgG), immunoglobulin M (IgM), interleukin 6 (IL-6), and tumor necrosis factor-α (TNF-α) in the supernatants were measured using commercial ELISA kits (Nanjing Jiancheng Institute of Bioengineering, Nanjing, Jiangsu, China) for chickens according to the manufacturer’s instructions. The activities of D-lactate (D-Lac) and diamine oxidase (DAO) in the serum samples were determined using available kits (Nanjing Jiancheng Institute of Bioengineering). The concentrations of total superoxide dismutase (SOD) and malondialdehyde (MDA) in the ileum and serum samples were quantified using commercial kits (Nanjing Jiancheng Institute of Bioengineering) following the manufacturer’s instructions.

### Quantitative Real-Time PCR Analysis

Total RNA from the ileum samples was extracted using RNAiso plus (TaKaRa Biotechnology, Dalian, China), and the concentration and purity of RNA were measured by a NanoDrop^Tm^ 2000 spectrophotometer (Thermo Fisher Scientific, MA, United States). Then, the RNA was reverse transcribed into complementary DNA (cDNA) using HiScript II Q RT SuperMix (Vazyme Biotech, Nanjing, China). Real-Time PCR was performed in a volume of 20 μl using TB Green Premix EX Taq^TM^ (TaKaRa Biotechnology) and the StepOnePlus Real-Time PCR System (Applied Biosystems, CA, United States). The thermal cycling conditions were as follows: 95°C for 5 min, followed by 40 cycles of 95°C for 10 s and 60°C for 30 s. The primers used are listed in Table 1. The relative messenger RNA (mRNA) expression levels of the target genes were calculated using the 2^–ΔΔCt^ method and were normalized to β-actin expression. Three independent experiments were carried out, and each experiment was repeated three times.

**TABLE 1:** The primers used in this study.

Primers	Forward	Reverse	References
341F-806R	CCTAYGGGRBGCASCAG	GGACTACNNGGGTATCTAAT	Tang et al., 2019
IL-6	AAATCCCTCCTCGCCAATCT	CCCTCACGGTCTTCTCCATAAA	This study
TNF-α	TGCTGTTCTATGACCGCC	CTTTCAGAGCATCAACGCA	Zhao X. et al., 2020
IFN-γ	CTGACGGTGGACCTATTATTGTAG	GTTTGATGTGCGGCTTTGA	Zhao X. et al., 2020
IL-1β	GTGAGGCTCAACATTGCGCTGTA	TGTCCAGGCGGTAGAAGATGAAG	Zhao X. et al., 2020
IL-8	TTGGAAGCCACTTCAGTCAGAC	GGAGCAGGAGGAATTACCAGTT	This study
TGF-β1	GCGCTGTACAACCAACACAA	TTCCGGCCCACGTAGTAAAT	This study
Claudin-1	GCAGATCCAGTGCAAGGTGTA	CACTTCATGCCCGTCACAG	This study
Occludin	TCGTGCTGTGCATCGCCATC	CGCTGGTTCACCCCTCCGTA	Zhao X. et al., 2020
ZO-1	GCGCCTCCCTATGAGGAGCA	CAAATCGGGGTTGTGCCGGA	Zhao X. et al., 2020
Muc2	ATTGTGGTAACACCAACATTCATC	CTTTATAATGTCAGCACCAACTTCTC	Zhao X. et al., 2020
Tff2	CTGAACAGCAATAACCACCC	TAATCCCCACAGAGACCACA	Zhao X. et al., 2020
β-actin	ATGGCTCCGGTATGTGCAA	TGGGCTTCATCACCAACGTA	This study

### Western Blot Analysis

The intestine samples were homogenized in radioimmunoprecipitation assay (RIPA) lysis buffer (Solarbio, Beijing, China) with phenylmethanesulfonyl fluoride added and were centrifuged at 12,000 × *g* for 5 min. The protein concentration in the supernatant was measured using a bicinchoninic acid protein assay kit (Bio-Platform, Shanghai, China), and then, the supernatant was resuspended in 5 × sodium dodecyl sulfate-polyacrylamide gel electrophoresis (5 × SDS-PAGE) sample loading buffer (Beyotime, Shanghai, China) and denatured for 10 min at 100°C before being separated by SDS-PAGE (at a constant voltage of 80 V for 30 min, then 120 V for 60 min). The proteins were transferred to nitrocellulose membranes (constant current of 300 mA for 60 min), which were blocked in 5% non-fat milk for 2 h at room temperature and then incubated with diluted primary antibodies against β-actin (1:5,000, Sunshine Bio, Nanjing, China), claudin-1 (1:500, Beyotime), occludin (1:500, Beyotime), and ZO-1 (1:500, Biorbyt Ltd., Cambridge, United Kingdom) overnight at 4°C. After three washes with 1 × PBS–Tween 20, the nitrocellulose membranes were incubated with horseradish peroxidase-labeled goat antimouse IgG (1:1,000, Beyotime) or horseradish peroxidase-labeled goat antirabbit IgG (1:1,000, Beyotime) for 6 h at 4°C, and the protein bands were visualized using an ECL detection system (Bio-Rad, Hercules, CA, United States). Three independent experiments were carried out, and each experiment was repeated three times.

### Intestinal Microbial Community Analysis

Intestinal digesta samples were collected from the Sanhuang chickens. Microbial DNA was extracted using the E.Z.N.A. <^®^ Stool DNA kit (Omega Bio-tek, Norcross, GA, United States). The 16S ribosomal RNA gene V3–V4 region was amplified by PCR using primers 341F and 806R. Thirty samples (*n* = 6/group) were barcoded and pair-end sequenced individually on the Illumina MiSeq platform (Shanghai Biozeron Co., Ltd., Shanghai, China). The sequencing results have been submitted to the Sequence Read Archive database of the NCBI (accession number: PRJNA757904). In this study, amplicon sequence variants (ASVs) were used in place of operational taxonomic units (OTUs). ASVs were created by grouping unique sequences and were equivalent to 100% OTUs. Raw fastq files were first demultiplexed using in-house Perl scripts, and then, parsed sequences were dereplicated and subjected to the DADA2 algorithm to identify indel-mutations and substitutions. Beta-diversity analysis was performed using the Bray–Curtis dissimilarity metric to compare the results of the principal coordinates analysis (PCoA). For the identification of biomarkers for highly dimensional intestinal bacteria, linear discriminant analysis effect size (LEfSe) was performed. A Kruskal–Wallis sum-rank test was performed to examine the changes and dissimilarities among classes followed by linear discriminant analysis (LDA) to determine the size effect of each distinctively abundant taxa.

### Statistical Analysis

The results are mainly presented as the mean ± SD from three independent experiments. Statistical differences in *Salmonella* growth curves among multiple groups were performed by a two-tailed paired-sample *t*-test. Statistical differences in the abundance of bacteria among multiple groups were determined by non-parametric factorial Kruskal–Wallis sum-rank test. Statistical differences in the PCoA analysis among multiple groups were identified using analysis of similarities (ANOSIM). Statistical differences in the LDA analysis among multiple groups were determined by linear regression analysis. Statistical differences in the other experiments among multiple groups were determined by one-way analysis of variance (ANOVA), followed by a Duncan multiple range test and Fisher’s least significant difference (LSD) *post hoc* test using the SPSS 16.0 statistical software (SPSS, Chicago, IL, United States). *p* < 0.05 and *p* < 0.01 were considered significant.

## Results

### Preparation and Anti-*Salmonella* Assay of the Cu/Zn-Pal Complexes

**FIGURE 1:**
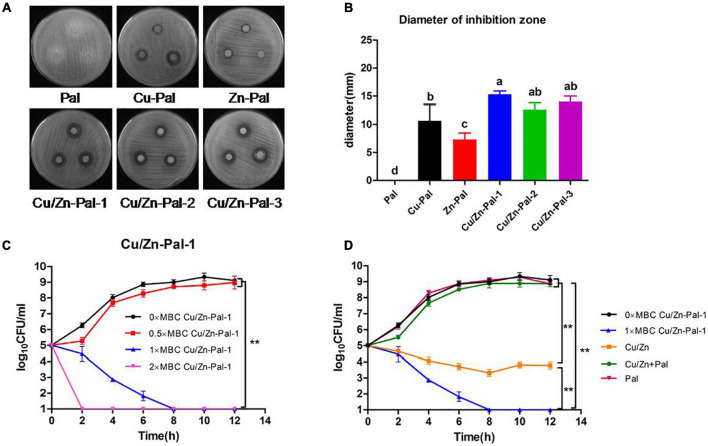
Cu/Zn-Pal complexes inhibit *S.* Typhimurium growth *in vitro*. **(A)** The agar diffusion assay results of the Pal, Cu-Pal, Zn-Pal, and Cu/Zn-Pal complexes. **(B)** The diameter of the inhibition zone for the Pal, Cu-Pal, Zn-Pal, and Cu/Zn-Pal complexes was detected (*n* = 9/group). Data are expressed as the mean ± SD. Significant differences were identified using one-way ANOVA statistical analysis. Different letters indicate that the changes between groups were statistically significant (*p* < 0.05). **(C)** The effect of different concentrations of Cu/Zn-Pal-1 on the *S.* Typhimurium growth curve (*n* = 9/group): 0 × MBC Cu/Zn-Pal-1 group, negative control without the addition of Cu/Zn-Pal-1; 0.5 × MBC, 1 × MBC, and 2 × MBC Cu/Zn-Pal-1 groups, addition of 0.5 × MBC, 1 × MBC, and 2 × MBC Cu/Zn-Pal-1, respectively. **(D)** The effect of different substances on the *S.* Typhimurium growth curve (*n* = 9/group): 0 × MBC and 1 × MBC Cu/Zn-Pal-1 groups are the same as the 0 × MBC and 1 × MBC Cu/Zn-Pal-1 groups, respectively, in **(C)**; Cu/Zn group, addition of CuSO_4_ and ZnCl_2_ in which the contents of Cu^2+^ and Zn^2+^ were equal to that in the 1 × MBC Cu/Zn-Pal-1 group; Cu/Zn + Pal group, addition of CuSO_4_ and ZnCl_2_ mixed with Pal in which the contents of Cu^2+^, Zn^2+^, and Pal were equal to those in the 1 × MBC Cu/Zn-Pal-1 group; Pal group, addition of natural Pal in which the content of Pal was equal to that in the 1 × MBC Cu/Zn-Pal-1 group. Data are expressed as the mean ± SD. Significant differences in *Salmonella* growth curves were analyzed using a two-tailed paired-sample *t*-test. **p < 0.01.

Cu/Zn-Pal complexes were prepared *via* ion-exchange reactions and then the copper and zinc contents in the complexes were measured. The results are listed in Table 2. The anti-*Salmonella* capacities of the complexes were also evaluated. An agar diffusion assay showed that there was no inhibition zone around Pal, whereas the Cu/Zn-Pal complexes and Cu-Pal showed a significant increase in the diameter of the inhibition zone (*p* < 0.05; Figures 1A,B). In addition, the MBCs of the Cu/Zn-Pal-1 and Cu-Pal complexes were lower than those of Pal and Zn-Pal. The detailed results of the MBC of each complex are listed in Table 3. The growth curve data revealed that *S.* Typhimurium proliferated rapidly in the 0 × MBC (negative control group without the addition of Cu/Zn-Pal-1) and 0.5 × MBC Cu/Zn-Pal-1 groups, while the growth of *S.* Typhimurium was significantly inhibited in the 1 × MBC and 2 × MBC Cu/Zn-Pal-1 groups (*p* < 0.05; Figure 1C). However, Figure 1D shows that mixing the three substances (Cu^2+^, Zn^2+^, and Pal) in non-complexed form did not significantly inhibit the growth of *Salmonella* compared with that of the 1 × MBC Cu/Zn-Pal-1 group.

**TABLE 2:** The Cu^2+^/Zn^2+^ concentration in the complexes.

Sample	Cu^2+^ (mg/g)	Zn^2+^ (mg/g)
Pal	/	/
Cu-Pal	51	/
Zn-Pal	/	16.23
Cu/Zn-Pal-1	48.23	3.1
Cu/Zn-Pal-2	36.5	8.83
Cu/Zn-Pal-3	15.7	14.2

**TABLE 3:** The minimum bactericidal concentration (MBC) of the different complexes.

Sample	MBC (mg/ml)
Pal	>64
Cu-Pal	7.0
Zn-Pal	>64
Cu/Zn-Pal-1	5.0
Cu/Zn-Pal-2	9.5
Cu/Zn-Pal-3	36

### Cu/Zn-Pal-1 Attenuates *S.* Typhimurium Infection in Chickens

**FIGURE 2:**
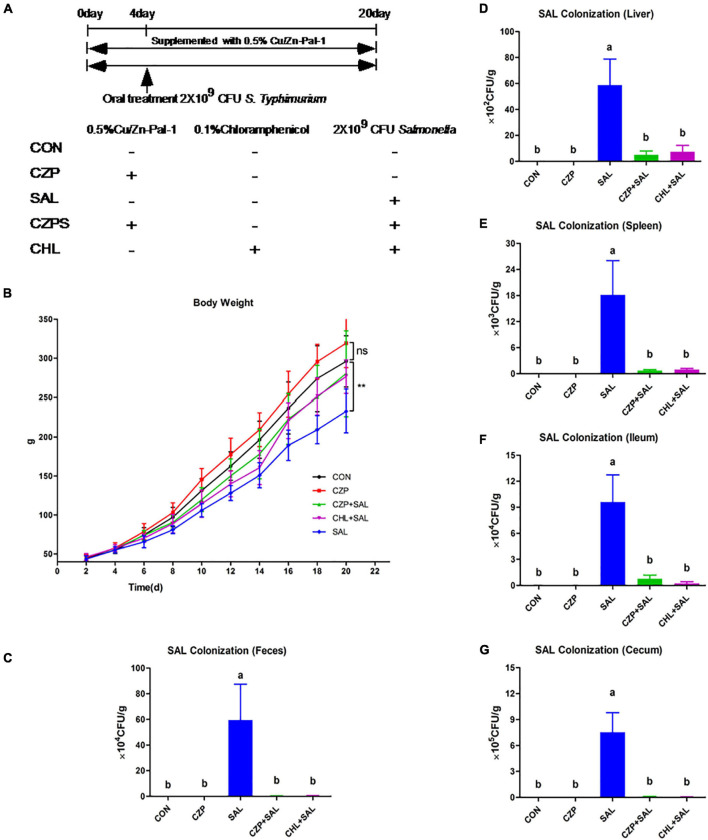
Cu/Zn-Pal-1 enhances weight gain and significantly reduces the *S.* Typhimurium load in the feces and tissues of infected chicks. **(A)** Schematic diagram of the animal experiment. **(B)** The changes in body weight of the chickens during the experiment (*n* = 15/group). Data are expressed as the mean ± SD. Differences in the body weights of each group of chickens at day 20 were analyzed by one-way ANOVA. **p < 0.01. ns, no significant difference. **(C–G)** The load of *S.* Typhimurium in feces, the liver, spleen, ceca, and ileum (*n* = 9/group). Data are expressed as the mean ± SD. Significant differences were detected using one-way ANOVA statistical analysis (*p* < 0.05). Different letters indicate that changes between groups are statistically significant.

To investigate whether Cu/Zn-Pal-1 pretreatment has a protective effect on chicks infected with *S.* Typhimurium, Sanhuang chickens were randomly allocated to five groups that were subjected to different pretreatments (Figure 2A). We found that the body weight of infected chickens at day 20 (233 g/chicken) was significantly lower than those in the CON group (296 g/chicken) (*p* < 0.05), while Cu/Zn-Pal-1 pretreatment in the CZPS group (280 g/chicken) significantly improved their body weight (*p* < 0.05; Figure 2B). Additionally, the survival and pathogenicity of *S.* Typhimurium can be evaluated by its colonization in feces and its translocation to organs, respectively (Xu et al., 2018). Therefore, we detected *S.* Typhimurium colonization in feces and tissues including the liver, spleen, ileum, and ceca. The data showed that *S.* Typhimurium colonization reached 10^5^.^7^ CFU/g in the feces of the SAL group on day 20, while Cu/Zn-Pal-1 significantly decreased the *S.* Typhimurium load in the CZPS group (10^3^.^2^ CFU/g; *p* < 0.05; Figure 2C). We also observed a high load of *Salmonella* in the tissues of infected chickens, while the load of *S.* Typhimurium was lower following Cu/Zn-Pal-1 and chloramphenicol pretreatment in the CZPS and CHL groups compared with the SAL group (*p* < 0.05; Figures 2D–G).

### Cu/Zn-Pal-1 Ameliorates *S.* Typhimurium-Induced Viscera Lesions and Intestinal Mucosa Damage

**FIGURE 3:**
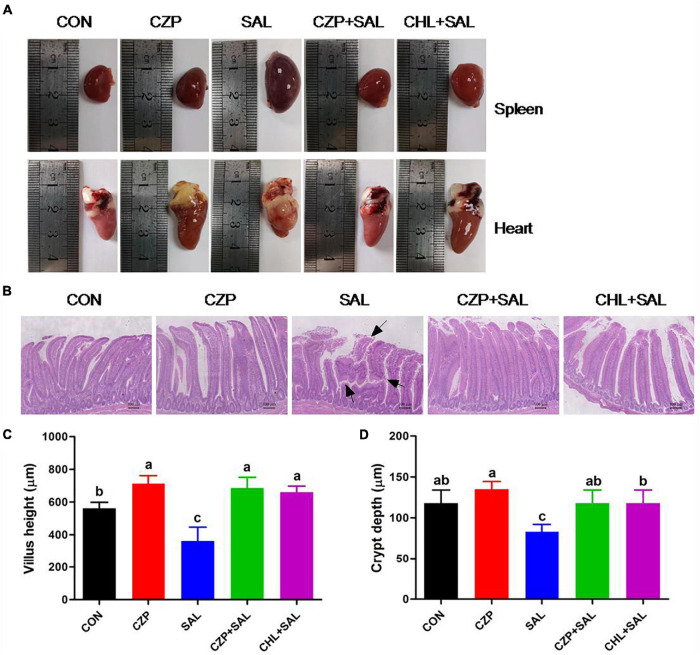
Cu/Zn-Pal-1 ameliorates *S.* Typhimurium-induced viscera lesions and intestinal mucosa damage. **(A)** Anatomical morphology of the spleen and heart in chickens from each group. **(B)** Histological examination of the duodenum mucosa. Scale bar, 100 μm. The areas marked with an arrow show villous shedding and intestinal mucosa injury induced by *S.* Typhimurium. **(C)** Villus length in the duodenum (*n* = 6/group). **(D)** Crypt depth in the duodenum (*n* = 6/group). Data are expressed as the mean ± SD. Significant differences were identified using one-way ANOVA statistical analysis (*p* < 0.05). Different letters indicate that the changes between groups are statistically significant.

As shown in Figure 3A, a purplish black color indicated edema of the spleen, and distinct lesions in the heart were evident in the *S.* Typhimurium-challenged group. By contrast, the spleen and heart appeared normal in morphology and structure in the CZPS and CHL groups. Histological examination exhibited an orderly arrangement of villi and crypts of the duodenum and a clear and integrated gland structure in the CON group, as was also observed in the CZP group. Compared with the uninfected control (CON) group, villous shedding was evident, and intestinal epithelial cells were damaged in the SAL group (Figure 3B). However, Cu/Zn-Pal-1 pretreatment in the CZPS group resulted in greater villus height and crypt depth compared with the SAL group (*p* < 0.05; Figures 3C,D). Moreover, the intestinal villus height in the CZP group following the addition of Cu/Zn-Pal-1 was higher than that in the CON group (*p* < 0.05; Figure 3C).

### Cu/Zn-Pal-1 Modulates the Expression Levels of Immune Cytokines in the Intestine

**FIGURE 4:**
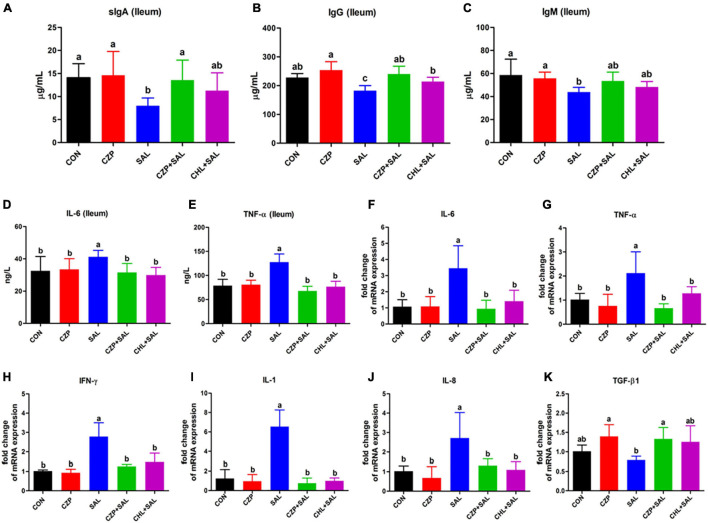
Cu/Zn-Pal-1 modulates the expression levels of immune cytokines in the intestine. **(A–C)** The protein expression levels of sIgA, IgG, and IgM in the ileum were detected using ELISA kits (*n* = 6/group). **(D,E)** The protein expression levels of IL-6 and TNF-α in the ileum were determined by ELISA kits (*n* = 6/group). **(F–K)** The mRNA expression levels of cytokines (IL-6, TNF-α, IFN-γ, IL-1, IL-8, and TGF-β1) in the ileum were detected by real-time PCR (*n* = 9/group). Data are expressed as the mean ± SD. Significant difference were identified using one-way ANOVA statistical analysis (*p* < 0.05). Different letters indicate that the changes between groups are statistically significant.

To explore the effects of Cu/Zn-Pal-1 on immune function in the infected chickens, the concentrations of sIgA, IgG, and IgM in the ileum were detected. The levels of sIgA, IgG, and IgM were found to be lower in the SAL group compared with those in the CON group, whereas pretreatment with Cu/Zn-Pal-1 recovered the levels of sIgA and IgG in the CZPS group (*p* < 0.05; Figures 4A,B). Cu/Zn-Pal-1 also increased the content of IgM, but there was no significant difference between the CZPS group and the SAL group (*p* > 0.05; Figure 4C). Additionally, pro- and anti-inflammatory cytokines play a key role in protecting chickens against *S.* Typhimurium infection. Therefore, we determined the protein and mRNA expression levels of cytokines using an ELISA assay and a quantitative real-time PCR assay, respectively. As shown in Figures 4D,E, the concentrations of IL-6 and TNF-α in the SAL group were obviously higher than those in the CON group (*p* < 0.05), which was consistent with the results obtained for the mRNA relative expression levels of IL-6 and TNF-α (Figures 4F,G). Furthermore, *S.* Typhimurium challenge resulted in upregulation of the mRNA expression levels of proinflammatory cytokines including interferon gamma (IFN-γ), IL-1, and IL-8, coupled with downregulation of the anti-inflammatory cytokine transforming growth factor beta 1 (TGF-β1) in the SAL group compared with those in the CON group (*p* < 0.05), while Cu/Zn-Pal-1 and chloramphenicol markedly reversed the mRNA relative expression of pro- and anti-inflammatory cytokines in the CZPS and CHL groups, respectively (Figures 4H–K).

### Cu/Zn-Pal-1 Ameliorates the *Salmonella*-Induced Permeability of the Intestinal Mucosal Barrier

**FIGURE 5:**
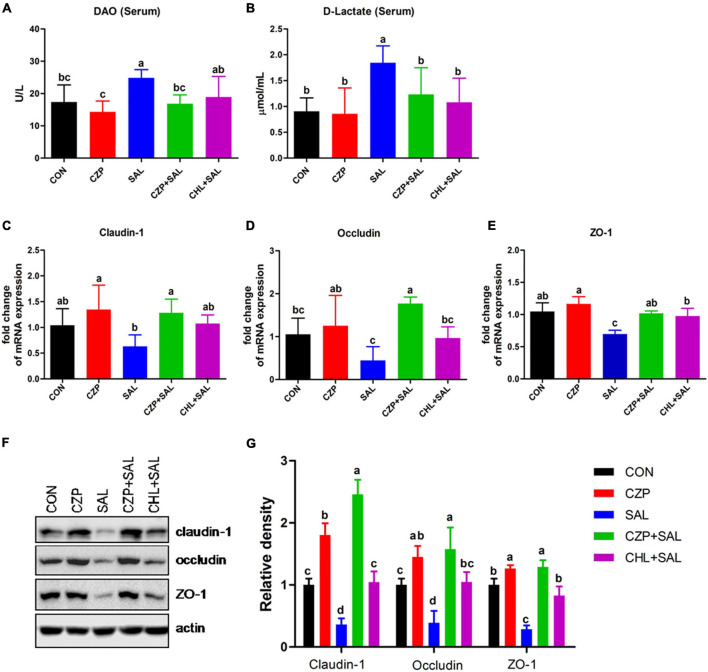
The effect of Cu/Zn-Pal-1 on intestinal barrier function. **(A,B)** Serum DAO and D-Lac contents were determined using kits (*n* = 6/group). **(C–E)** The mRNA expression levels of claudin-1, occludin, and ZO-1 in the intestine of chicks were detected by real-time PCR (*n* = 9/group). **(F,G)** The protein expression levels of claudin-1, occludin, and ZO-1 in the intestine of chicks were tested by Western blotting (*n* = 3/group). Data are expressed as the mean ± SD. Significant differences were identified using one-way ANOVA statistical analysis (*p* < 0.05). Different letters indicate that the changes between groups are statistically significant.

**FIGURE 6:**
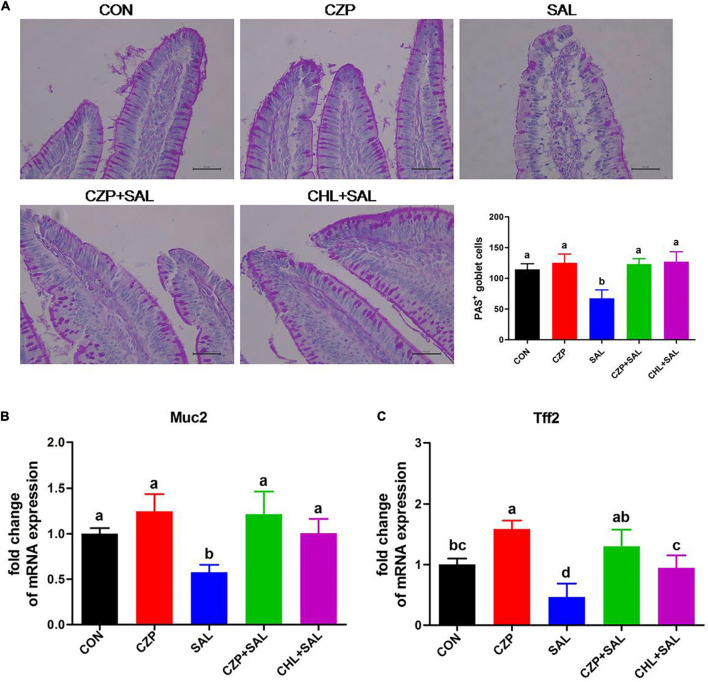
Cu/Zn-Pal-1 promotes the production of goblet cells and upregulates the expression levels of related genes. **(A)** Periodic acid–Schiff staining of the duodenum sections (*n* = 6/group). Goblet cells were counted for each villus. Scale bar 100 μm. **(B,C)** Muc2 and Tff2 mRNA expression levels in the duodenum of the chicks were detected by qRT-PCR (*n* = 9/group). Data are expressed as the mean ± SD. Significant differences were detected using one-way ANOVA statistical analysis (*p* < 0.05). Different letters indicate that the changes between groups are statistically significant.

The intestinal epithelial barrier plays a vital role in preventing penetration of pathogens. In this study, we assessed the effects of Cu/Zn-Pal-1 on intestinal mucosal barrier function by detecting the contents of DAO and D-Lac in serum. As expected, the concentrations of DAO and D-Lac in the SAL group were significantly higher than those in the CON group (*p* < 0.05). However, pretreatment with Cu/Zn-Pal-1 significantly reduced the levels of DAO and D-Lac in the CZPS group relative to the SAL group (*p* < 0.05; Figures 5A,B), as also observed in the CHL group. In addition, the tight junction proteins are a major component of the intestinal mucosal barrier. We also determined the mRNA expression levels of tight junction proteins. We found upregulation of the mRNA relative expression of claudin-1, occludin, and ZO-1 following pretreatment with Cu/Zn-Pal-1 alone, but these were not statistically significant differences from the CON group (*p* > 0.05; Figures 5C–E). Western blot analysis demonstrated that the protein expression levels of claudin-1, occludin, and ZO-1 were significantly increased following pretreatment with Cu/Zn-Pal-1 alone in the CZP group compared with those in the CON group (*p* < 0.05). However, *S.* Typhimurium infection dramatically decreased the expression levels of tight junction proteins in the SAL group relative to the CON group (*P* < 0.05), but Cu/Zn-Pal-1 addition significantly increased their expression levels in the CZPS group compared with the SAL group (*p* < 0.05; Figures 5F,G). Mucus, mainly secreted by goblet cells, plays an important role in intestinal mucosal barrier function. Periodic acid–Schiff staining revealed that *S.* Typhimurium infection resulted in a greater reduction of goblet cells in the SAL group compared with the CON group (*p* < 0.05). While compared with the SAL group, Cu/Zn-Pal-1 and chloramphenicol pretreatment increased the production of goblet cells after *S.* Typhimurium challenge (*p* < 0.05; Figure 6A). Furthermore, the mRNA relative expression levels of Muc2 and Tff2 were detected by real-time PCR. We found that the mRNA expression levels of these two genes in the SAL group were lower than in the CON group (*p* < 0.05). However, Cu/Zn-Pal-1 significantly upregulated the mRNA expression of Muc2 and TFF2 in the CZPS group compared with the SAL group (*p* < 0.05; Figures 6B,C).

### Cu/Zn-Pal-1 Attenuates the *S.* Typhimurium-Induced Oxidative Stress Response

**FIGURE 7:**
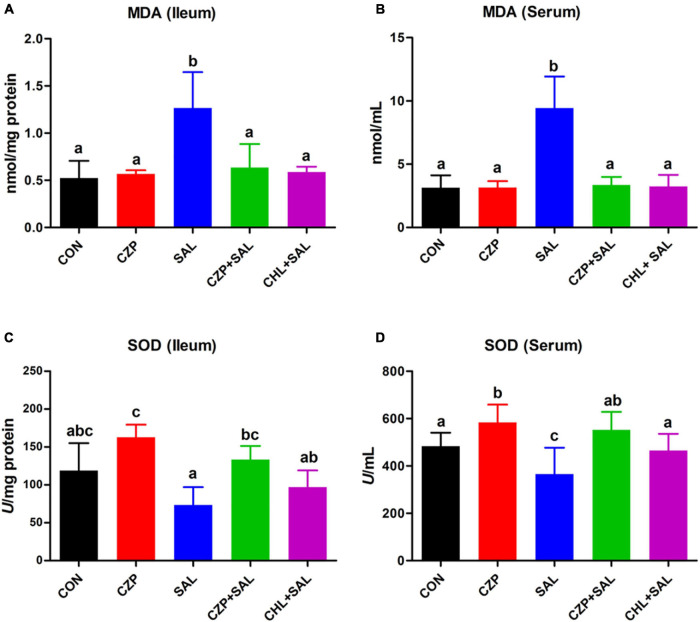
Cu/Zn-Pal-1 attenuates the *S.* Typhimurium-induced oxidative stress response. **(A,B)** The MDA contents in the ileum and serum were detected using kits (*n* = 9/group). **(C,D)** The SOD contents in the ileum and serum were detected using kits (*n* = 9/group). Data are expressed as the mean ± SD. Significant differences were detected using one-way ANOVA statistical analysis (*p* < 0.05). Different letters indicate that the changes between groups are statistically significant.

The antioxidant defense system plays a significant role in eliminating oxygen free radicals and protecting cells and organs from free radical-associated damage (Cheng et al., 2016). In this study, we investigated the effects of Cu/Zn-Pal-1 on the *S.* Typhimurium-induced oxidative stress response. The MDA content usually reflects the degree of lipid peroxidation and indirectly reflects the degree of cell damage (Su et al., 2018). Not surprisingly, after *Salmonella* challenge, the MDA contents were significantly increased in the ileum and serum compared with that of the CON group (*p* < 0.05), whereas Cu/Zn-Pal-1 and chloramphenicol pretreatment reversed these effects and decreased the MDA contents (Figures 7A,B). The SODs are the most effective component of the antioxidant enzyme defense system against reactive oxygen species (Zelko et al., 2002). Compared with the SAL group, the SOD contents in the CZPS and CHL groups were also significantly enhanced (*p* < 0.05; Figures 7C,D). The above results confirmed that the anti-*Salmonella* properties of Cu/Zn-Pal-1 may reduce the amount of *Salmonella* and indirectly alleviate the *S.* Typhimurium-induced oxidative stress response.

### Cu/Zn-Pal-1 Modulates the Intestinal Microbiota of Chickens

**FIGURE 8:**
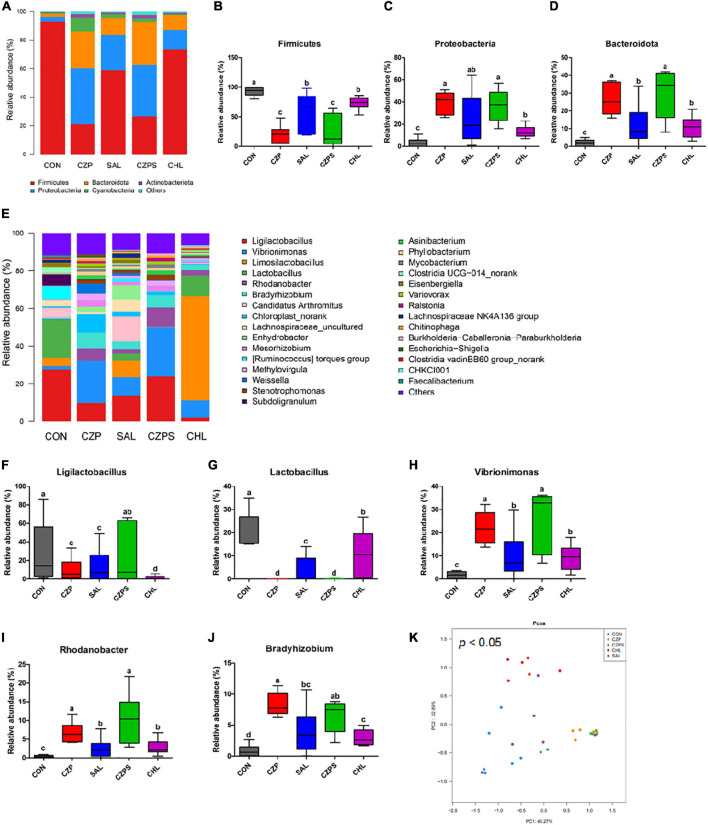
Compositional analysis and principal coordinates analysis of the intestinal microbiota (*n* = 6/group). **(A)** Relative abundance of members of the intestinal microbiota at the phylum level. **(B–D)** Relative abundance of Firmicutes, Proteobacteria, and Bacteroidetes in chicken intestinal digesta samples. **(E)** Relative abundance of intestinal microbiota at the genus level. **(F–J)** Relative abundance of *Ligilactobacillus*, *Lactobacillus*, *Vibrionimonas*, *Rhodanobacter*, and *Bradyrhizobium* in chicken intestinal digesta samples. Significant differences were identified using non-parametric factorial Kruskal–Wallis sum-rank test (*p* < 0.05). Different letters indicate that the changes between groups are statistically significant. **(K)** Principal coordinates analysis (PCoA). Significant differences were identified using ANOSIM analysis (*p* < 0.05).

**FIGURE 9:**
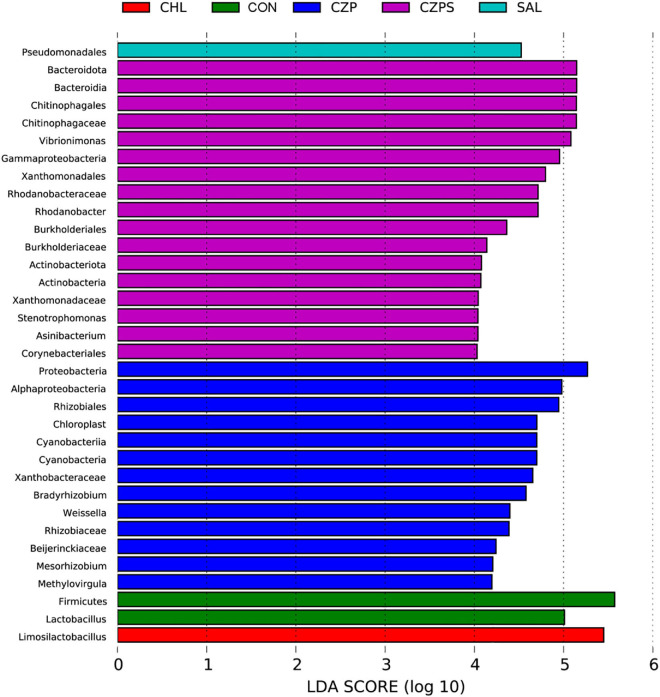
The taxa of the intestinal microbiota that varied most significantly in abundance were identified using LDA combined effect size measurements (*n* = 6/group) (*p* < 0.05, log LDA score > 4).

To investigate the regulatory role of Cu/Zn-Pal-1 on the intestinal microbiota in chickens, we performed 16S ribosomal RNA gene sequencing of samples from the intestinal digesta (*n* = 6/group). The compositional analytic results showed that Firmicutes, Proteobacteria, Bacteroidetes, Cyanobacteria, and Actinobacteria were the most dominant bacteria in all of the groups at the phylum level (Figure 8A). Compared with the CON group, *Salmonella* challenge dramatically reduced the relative abundance of Firmicutes and increased the abundance of Proteobacteria and Bacteroidetes (*p* < 0.05). However, Cu/Zn-Pal-1 supplementation significantly decreased the abundance of Firmicutes and increased the proportion of Bacteroidetes in the CZP and CZPS groups compared with the SAL group (*p* < 0.05; Figures 8B–D). At the genus level, the relative abundance of intestinal microbiota is displayed in Figure 8E. *S.* Typhimurium administration significantly decreased the proportion of *Ligilactobacillus* and *Lactobacillus* from the SAL group compared with the CON group (*p* < 0.05), whereas Cu/Zn-Pal-1 supplementation in the CZPS group significantly increased the abundance of *Ligilactobacillus* (*P* < 0.05; Figure 8F), and chloramphenicol addition in the CHL group significantly enhanced the proportion of *Lactobacillus* compared with the SAL group (*p* < 0.05; Figure 8G). Furthermore, *S.* Typhimurium administration significantly enhanced the relative abundance of *Vibrionimonas*, *Rhodanobacter*, and *Bradyrhizobium* compared with those in the CON group (*p* < 0.05). There was no significant difference in the relative abundance of these three bacterial genera between the CHL group and the SAL group (*p* > 0.05). However, the supplementation with Cu/Zn-Pal-1 in the CZP and CZPS groups significantly increased the relative abundance of these three bacterial genera compared with the SAL group (*p* < 0.05; Figures 8H–J). Besides, PCoA revealed that there was a significant difference between groups overall using ANOSIM analysis (*p* < 0.05), and microbiota composition of each group apart from the SAL group showed a distinct clustering (Figure 8K). The LEfSe analytic results revealed differences in the abundance of taxa in these groups (*p* < 0.05, LDA > 4.0). The relative abundances of Firmicutes and *Lactobacillus* were enriched in the CON group, whereas the relative proportion of Pseudomonadales was significantly increased with *S.* Typhimurium challenge. Bacteroidetes, Burkholderiales, and Actinobacteria were mainly enriched in the CZPS group. In the CHL group, the relative abundance of *Limosilactobacillus* was significantly increased (Figure 9). Collectively, these data demonstrate that Cu/Zn-Pal-1 may modulate the gut microbiota of chickens to some extent.

## Discussion

Clay nanoparticles are usually used as carriers for drug delivery, to carefully control the release and action of a range of drugs (Mousa et al., 2018; Sabzevari et al., 2020). Compared with montmorillonite and other antibacterial materials, Pal not only has stronger absorptive capacity and greater chemical stability, but it also has richer reserves and a lower price (Li et al., 2014). Therefore, modified Pals are widely used in the veterinary field. A recent study found that dietary Pal addition could decrease lead residues in the tissues of broilers and alleviate oxidative stress (Cheng Y. F. et al., 2018). In addition, the supplementation of quaternary ammonium derivative N-(2-hydroxy) propyl-3-trimethyl ammonium chito-oligosaccharide chloride modified Pal ameliorated adverse effects in chickens challenged with a low level of *Fusarium* mycotoxin contamination (Cheng Y. et al., 2018). In poultry farms, severe salmonellosis outbreaks occur frequently in chicken flocks and result in huge economic losses. Moreover, *Salmonella* is transmitted from contaminated poultry products to humans *via* the food chain, with a risk of acute gastroenteritis, diarrhea, and even death in humans. Therefore, it is necessary to reduce or even eliminate *Salmonella* infection in chickens. In this study, we found that Cu/Zn-Pal-1 supplementation reduces *Salmonella* colonization, protects the intestinal mucosal barrier, and impedes the development of disease in chickens after *S.* Typhimurium challenge.

The inhibition ability of bacteria can be estimated by a susceptibility assay *in vitro* (Jiang et al., 2019; Gámez et al., 2020), and a bacterial growth curve forms the basis of the susceptibility test (Xu H. et al., 2020). The current study showed that there was no inhibition zone around natural Pal, which is consistent with a previous study that natural clay minerals have no antibacterial activity (Rapacz-Kmita et al., 2017). Cu/Zn-Pal-1 in complexed form effectively inhibited the growth of *Salmonella*, but the three substances (Cu^2+^, Zn^2+^, and Pal) in non-complexed form showed no significant effects. This suggested that combining the ion carrier properties of Pal with the major antibacterial properties of copper ions resulted in higher antibacterial capacity. Moreover, the antibacterial properties of Cu/Zn-Pal-1 may be superior to those of GEO/Pal because a previous study reported that the minimum inhibitory concentration of GEO/Pal to inhibit the growth of *E. coli* was 25 mg/ml (Lei et al., 2017), whereas the MBC of Cu/Zn-Pal-1 to inhibit the growth of *S.* Typhimurium was 5 mg/ml.

A large body of evidence suggests that *Salmonella* challenge decreases productivity and increases the *Salmonella* colonization of organs (Symonds et al., 2012; Zhang H. et al., 2020). In our study, compared with the uninfected control (CON) group, the body weight of chickens infected with *Salmonella* was significantly decreased, and the *S.* Typhimurium load was dramatically increased in the feces and organs, which was consistent with a previous report (Zhang H. et al., 2020). However, compared with the group infected with *Salmonella* (SAL), Cu/Zn-Pal-1 supplementation in the CZPS group enhanced weight gain in the infected chickens and significantly attenuated the *S.* Typhimurium load in feces and tissues. These results suggested that Cu/Zn-Pal-1 pretreatment does lower the pathogenic load following *Salmonella* infection *in vivo* and reduces the salmonellosis burden, thereby enhancing weight gain in the challenged chickens.

The small intestine is rich in microvilli and is the primary site of nutrient absorption (Zhang L. et al., 2020). However, once attached to intestinal epithelial cells, salmonellae assemble the type III secretion system and transport effectors into intestinal cells, which results in rearrangement of the actin cytoskeleton, the disruption of tight junctions, and subsequent *Salmonella* invasion (Haraga et al., 2008; Foley et al., 2013; Larock et al., 2015). Finally, salmonellae destroy the intestinal cell surface microvilli and then impede the intestinal absorption function (Zhang L. et al., 2020). Our current results confirmed that *Salmonella* induces disease in the organs of chickens. However, Cu/Zn-Pal-1 showed similar drug efficacy to chloramphenicol in restoring the pathological organs and injured intestinal epithelial cells to normal morphology after *S.* Typhimurium challenge. Moreover, Cu/Zn-Pal-1 addition enhanced villus height in the intestines. Taken together, Cu/Zn-Pal-1 may effectively reduce *S.* Typhimurium colonization in the intestine and ameliorate *Salmonella*-induced intestinal mucosa damage while promoting the growth of intestinal villi to a certain extent.

Immunoglobulins sIgA, IgG, and IgM, mainly produced by B lymphocytes, exist in the mucosal immune system of the intestine as effector molecules to exert humoral immunity (Yuan et al., 2019; Yu et al., 2020). One study showed that modified Pal supplementation increased the immunoglobulin contents in broilers (Su et al., 2018). However, *Salmonella* infection induces a strong inflammatory response (Li et al., 2013; Stefan et al., 2017), and this excessive inflammation may be associated with gut permeability and subsequent dissemination of *Salmonella* (Mcarthur et al., 2015). In this study, we provide evidence that Cu/Zn-Pal-1 addition may reduce *Salmonella* load and indirectly modulate the levels of immune cytokines to avoid the excessive inflammatory response induced by *S.* Typhimurium infection.

The intestinal mucosal barrier plays a pivotal role in the selective infiltration of water, nutrients, endotoxin, and pathogens (Yang et al., 2019; Guerre, 2020). DAO and D-Lac, markers of intestinal epithelial barrier function, enter the bloodstream when the integrity of the intestinal mucosal barrier is compromised (Cheng et al., 2019; Zhang N. et al., 2020). In the present study, we found that Cu/Zn-Pal-1 pretreatment significantly decreased the DAO and D-Lac contents in the CZPS group compared with the SAL group. Tight junction proteins (e.g., claudin-1, occludin, and ZO-1) are crucial molecules for the maintenance of intestinal epithelial barrier integrity (Pei et al., 2019; Konieczka et al., 2020). Previous studies reported that *S.* Typhimurium could disrupt the tight junction structure in broilers and T84 intestinal epithelial cells (Zhang H. et al., 2020). Our results showed that the expression levels of tight junction proteins were significantly downregulated with *S.* Typhimurium challenge relative to the CON group, which was consistent with previous reports (Zhang H. et al., 2020). However, pretreatment with Cu/Zn-Pal-1 attenuated the intestinal epithelial barrier damage induced by *S.* Typhimurium through the modulation of tight junction protein expression. In addition, we found that supplementation with Cu/Zn-Pal-1 alone enhanced the expression levels of tight junction proteins in the CZP group relative to the CON group. Goblet cells, special intestinal epithelial cells in the gut, synthesize and secret mucins, such as Muc2, which are an important component of the intestinal mucosal barrier (Yin et al., 2019). The current findings suggest that *S.* Typhimurium infection reduces the number of goblet cells, accompanied by downregulation of the mRNA expression levels of Muc2 and Tff2, which is also consistent with a previous report (Zhu et al., 2020). However, Cu/Zn-Pal-1 addition may enhance the production of goblet cells and upregulate the mRNA expression of Muc2 and Tff2 in the CZPS group compared with the CON group. Taken together, these findings demonstrate that Cu/Zn-Pal-1 may reduce *Salmonella* colonization and impede salmonellosis progression, thereby ameliorating *Salmonella*-induced intestinal mucosal barrier damage, while Cu/Zn-Pal-1 may also stimulate the expression of tight junction proteins, promote the secretion of mucus and upregulate the expression of related genes to reinforce the integrity of the first line of defense in the gut.

Homeostasis of the intestinal microbiota plays a pivotal role in maintaining the biological barrier of the intestine, which prevents the invasion of pathogens from the intestinal tract into the organism (Paci et al., 2011; Zhao H. et al., 2020). In this study, to better understand the mechanism by which Cu/Zn-Pal-1 ameliorates *Salmonella-*induced intestinal dysfunction in chickens, we explored the effects of Cu/Zn-Pal-1 on the gut microbiota of chickens. At the phylum level, Firmicutes, Proteobacteria, and Bacteroidetes were the most dominant bacteria in the CON group, which was consistent with previous reports (He et al., 2020; Khan et al., 2020). *Salmonella* administration significantly decreased the abundance of Firmicutes and increased the relative abundance of Proteobacteria and Bacteroidetes compared with the CON group. It was consistent with previous reports that the proportion of Proteobacteria was also significantly increased, and the abundance of Firmicutes was reduced after *Salmonella* challenge (Chang et al., 2019). However, Cu/Zn-Pal-1 addition significantly improved the relative abundance of Bacteroidetes in the CZP and CZPS groups compared with the SAL group. Previous studies reported that dietary supplementation with Pal also increased the abundance of Bacteroidetes in the ceca of laying pullets compared with the control group and had beneficial effects on their growth (Chalvatzi et al., 2016). Bacteroidetes is not only involved in polysaccharide decomposition and the generation of short-chain fatty acids, which assist in energy harvesting by the host, but also directs the synthesis of mucosal glycans, which could offer great protection against the epithelial damage induced by a high-fat diet (Postler and Ghosh, 2017; Yang et al., 2020). Therefore, it demonstrates that an increased abundance of Bacteroidetes in the CZPS groups may contribute to alleviate *Salmonella*-induced intestinal epithelial damage.

At the genus level, *Salmonella* challenge significantly decreased the relative abundance of *Ligilactobacillus* and *Lactobacillus* compared with the CON group, which may make it easier for *Salmonella* to colonize the gut. A previous study reported that *Salmonella* challenge also decreased the population of *Lactobacillus* in the ceca (Chang et al., 2020). However, Cu/Zn-Pal-1 supplementation in the CZPS group significantly enhanced the proportion of *Ligilactobacillus* compared with the SAL group; it seems that Cu/Zn-Pal-1 reduces the *S.* Typhimurium load in the gut to avoid the effects of *Salmonella* on these taxa. Besides, it has been previously reported that *Ligilactobacillus* has immunomodulatory capabilities and protects chickens against *E. coli* O78 colonization (Masumizu et al., 2019; Wang et al., 2020). This suggests that the increase in the relative abundance of *Ligilactobacillus* in the CZPS group may further protect chickens against *Salmonella* colonization in the gut. In addition, previous studies reported that a region of the *Rhodanobacter* genome harbors a wide range of metal resistance genes, and these genes may be horizontally transferred (Cardenas et al., 2010; Hemme et al., 2010). In our study, Cu/Zn-Pal-1 pretreatment significantly increased the relative abundance of *Rhodanobacter* in the CZP and CZPS groups compared with the other three groups, which may be associated with the addition of copper and zinc ions. Previous preliminary experiments demonstrated that *S.* Typhimurium NJS1 stain was highly susceptible to chloramphenicol, so we set up the CHL group. In this study, the effects of chloramphenicol addition on intestinal microbiota were different from that of Cu/Zn-Pal-1. The addition with chloramphenicol significantly reduced the abundance of *Vibrionimonas*, *Rhodanobacter*, and *Bradyrhizobium* in the CHL group compared with the CZPS group. Previous studies reported that antibiotic therapy increased the abundance of Lactobacillales in chicken fecal microbiota (Videnska et al., 2013). We found that chloramphenicol addition also significantly increased the abundance of *Lactobacillus* in the CHL group compared with the CZPS group.

Linear discriminant analysis effect size analysis revealed the taxa of the gut microbiota that varied most significantly in abundance among groups. Notably, chloramphenicol supplementation significantly increased the relative abundance of *Limosilactobacillus* in the CHL group. *Limosilactobacillus* is a normal inhabitant of the animal gut and has been found to have anti-inflammatory effects (Lee et al., 2016; Diez-Echave et al., 2021), which may indicate that the increase in the abundance of *Limosilactobacillus* can attenuate the *S.* Typhimurium-induced excessive inflammatory response in the gut. In the CZPS group, several abundant species were identified, such as Bacteroidia, Burkholderiaceae, and Actinobacteria. Previous studies reported that the addition of a probiotic increased the abundance of Burkholderiaceae in the gut, along with an improved intestinal structure and growth performance in chickens (Li et al., 2019), which may imply that members of the Burkholderiaceae are beneficial to the gut health of chickens. Actinobacteria is a constituent of the normal gut microbiota and is reported to produce important secondary metabolites (enzymes and antibiotics), which play a key role in maintaining the health of the animal gut (Chen et al., 2017). Therefore, we suspected that Cu/Zn-Pal-1 may modulate the intestinal microbiota of chickens to a certain extent.

In summary, Cu/Zn-Pal-1 could effectively inhibit *S.* Typhimurium growth *in vitro*. Cu/Zn-Pal-1 also attenuated *S.* Typhimurium colonization *in vivo* and reversed the negative effects of infection in chickens. Furthermore, Cu/Zn-Pal-1may protect the intestinal mucosal barrier of chickens and modulate the intestinal microbiota. Therefore, the Cu/Zn-Pal-1 complex is proposed as a potentially effective feed supplement in reducing *S.* Typhimurium infection.

## Data Availability Statement

The datasets presented in this study can be found in online repositories. The names of the repository/repositories and accession number(s) can be found below: https://www.ncbi.nlm.nih.gov/, PRJNA757904.

## Ethics Statement

The animal study was reviewed and approved by the Institutional Animal Care and Use Committee of Nanjing Agricultural University.

## Author Contributions

CZ, DWY, and DJY conceived and designed the study. CZ, ZS, and YL performed the animal experiments. CZ, HC, and YG analyzed the data. CZ and PH wrote and revised the manuscript. All authors contributed to the article and approved the submitted version.

## Conflict of Interest

The authors declare that the research was conducted in the absence of any commercial or financial relationships that could be construed as a potential conflict of interest.

## Publisher’s Note

All claims expressed in this article are solely those of the authors and do not necessarily represent those of their affiliated organizations, or those of the publisher, the editors and the reviewers. Any product that may be evaluated in this article, or claim that may be made by its manufacturer, is not guaranteed or endorsed by the publisher.
